# Do patients infected with human coronavirus before the COVID-19 pandemic have less risk of being infected with COVID-19?

**DOI:** 10.55730/1300-0144.5846

**Published:** 2024-03-11

**Authors:** Gamze ŞANLIDAĞ İŞBİLEN, Ayça Aydın UYSAL, Selin YİĞİT, Özgür APPAK, Hilal SİPAHİ, Gülendam BOZDAYI, Arzu SAYINER, Candan ÇİÇEK, Özlem GÜZEL TUNÇCAN, Oğuz Reşat SİPAHİ

**Affiliations:** 1 Department of Infectious Diseases and Clinical Microbiology, Faculty of Medicine, Ege University, İzmir, Turkiye; 2 Department of Medical Microbiology, Faculty of Medicine, Ege University, İzmir, Turkiye; 3 Department of Medical Microbiology, School of Medicine, Division of Medical Virology, Gazi University, Ankara, Turkiye; 4 Department of Medical Microbiology, Faculty of Medicine, Dokuz Eylül University, İzmir, Turkiye; 5 Bornova Directorate of Health, İzmir, Turkiye; 6 Department of Infectious Diseases and Clinical Microbiology, Faculty of Medicine, Gazi University, Ankara, Turkiye; 7 King Hamad University Hospital, Bahrain Oncology Center, Department of Infectious Diseases, Al Muharraq, Bahrain

**Keywords:** HCoV, COVID-19, OC43, multiplex PCR, prevention, epidemiology

## Abstract

**Background/aim:**

Although seasonal human coronaviruses (HCoVs) have long been recognized as respiratory tract viruses, the newly identified SARS-CoV-2 caused a pandemic associated with severe respiratory failure. We aimed to evaluate the incidence of COVID-19 infection in patients diagnosed in three tertiary teaching hospitals, both with and without prior confirmed HCoV infection, and to compare these cohorts in terms of COVID-19 contraction.

**Materials and methods:**

In our study, we examined HCoV PCR-positive cases obtained retrospectively between January 2014 and March 2020 from three University Hospital Microbiology Laboratories (Cohort 1), as well as PCR-negative patients detected in the same PCR cycle as the positive cases (Cohort 2). We also evaluated subgroups of HCoV-positive cases.

**Results:**

There was no difference in COVID-19 contraction rates between Cohort 1 and Cohort 2 (p = 0.724). When previous HCoV subgroups of COVID-19-positive patients were examined, no significant difference was found between the betacoronavirus and alphacoronavirus subgroups (p = 0.822), among the four groups (NL63, 229E, OC43, HKU-1) (p = 0.207), or between the OC43 subgroup and the other groups (p = 0.295).

**Conclusion:**

Being previously infected with HCoV did not provide protection against COVID-19 in our study group. We suggest evaluating the possible effect of previous OC43 infection on COVID-19 contraction in larger cohorts.

## 1. Introduction

Human coronaviruses (HCoV) are large enveloped, positive-stranded RNA viruses divided into four groups. The globally endemic subtypes are HCoV 229E, NL63, OC43, and HKU1 [1]. These viruses are called non-SARS CoV. They may cause up to 1/3 of adult community-acquired upper respiratory tract infections. However, SARS-CoV or MERS-CoV present often with acute respiratory distress syndrome (ARDS) and both cause epidemics with high mortality [2].

The SARS-CoV-2 (COVID-19) virus, which was first identified in December 2019, spread rapidly across the world [3]. COVID-19 has a spectrum of asymptomatic infection to mild/moderate pneumonia and/or severe respiratory syndrome with fatal outcomes [1,4,5]. All HCoV, including SARS-CoV-2, may activate both innate and adaptive immune responses in infected patients. Theoretically, the antigenic similarity (as well as antibodies or immunity to those antigens) between COVID-19 and other HCoVs may cause cross-protection. Herein, we aimed to evaluate the incidence of COVID-19 infection in patients with and without prior confirmed HCoV infection.

## 2. Materials and methods

This retrospective cohort study, reported according to STROBE Criteria^1^, was conducted in three different tertiary-care educational hospitals located in two different cities with populations of 4,320,519^2^ and 5,663,322^3^.

In this study we compared the incidence of COVID-19 in two different cohorts:

Cohort 1 inclusion criterion was to be diagnosed with HCoV infection by respiratory specimen PCR (polymerase chain reaction) between January 2014 and March 10, 2020, (the first COVID-19 case in Türkiye was seen on March 11, 2020) in the study centers. Exclusion criteria were dying before the COVID-19 outbreak and being under 18 years of age (Figure).

Cohort 2 inclusion criterion was to have negative PCR detected in the same PCR cycle of cases in Cohort 1 in the study centers. Whenever possible, each Cohort 1 patient was matched with one Cohort 2 patient. Exclusion criteria were dying before the COVID-19 outbreak and being under 18 years of age (Figure).

COVID-19 contraction data of the Cohorts 1 and 2 were retrieved from the National Hospital Health Management System^4^ on December 10, 2020. We chose the December 10, 2020, as the cut off analysis time since COVID-19 vaccination started by that date.

We further performed a subgroup analysis to evaluate the possible effect of more recent immune response. In order to evaluate the more recent immune response, COVID-19 contraction results of 112 patients, who were found to be HCoV-positive between March 2019 and March 2020, were compared with control cases corresponding to the definition in Cohort 2.

This study was approved by the Scientific Research Platform of the Ministry of Health (2021-04-29T00_04_05) and Ege University Medical Research Institutional Review Board (2023-1200 23-7.1T/7).

Since there were no similar studies asking the same research question at the time we planned and collected the study data, we could not calculate the sample size, but a retrospective power analysis was performed^5^. Considering the number of exposed individuals (304), the risk of disease among the exposed (8.9%), the number of non-exposed individuals (297), and the risk of disease among the non-exposed (8.1%), the study’s power was determined to be 5%, which may be considered low.

IBM SPSS, version 25 for Windows was used for statistical analysis. Comparisons were made with chi-squared test. A (two-sided) p-value lower than 0.05 was accepted as statistically significant.

## 3. Results

A total of 776 adult cases fulfilled the criteria to be included in Cohort 1 or 2. However, 175 cases were excluded from the study because they died before the COVID-19 pandemic.

Cohort 1 comprised 304 HCoV-positive patients (159 females, aged 47.97 ± 18.13) and Cohort 2 included 297 negative control cases (145 females, aged 49.96 ± 19.03). Age and sex did not differ significantly between the cohorts (Table 1) (Figure).

When the subgroups of HCoV-positive patients in Cohort 1 were examined, the alphacoronavirus (229E, NL63, 229E/NL63) rate was 61.8% (n = 188) while the betacoronavirus (OC43, HKU1) rate was 31.9% (n = 97). The subgroup was not determined in 6.3% (n = 19) (Table 1). Overall, 8.9% (n = 27) of Cohort 1 were found to be COVID-19 PCR-positive. When the HCoV subgroups of the patients with positive COVID-19 PCR were evaluated, 63% (n = 17) were alphacoronavirus, 29.6% (n = 8) were betacoronavirus, and 7.4% (n = 2) did not have any subgroups (Table 2).

Cohort 2 comprised 297 patients, serving as the comparison group, in which negative control cases were not consistently present across all PCR cycles. Overall, 8.1% (n = 24) of them had COVID-19 PCR positivity. There was no difference between Cohorts 1 and 2 (p = 0.724, Table 1).

When previous HCoV subgroups of COVID-19-positive patients were examined, no significant difference was found between the betacoronavirus and alphacoronavirus subgroups (p = 0.822, Table 2) and between the four groups, i.e. NL63, 229E, OC43, HKU-1 (p = 0.211, Table 2) and OC43 subgroup vs others (5.2% [3/58] vs. 10.6% [22/227], p = 0.277, Table 2). Although there was no statistically significant difference between coronavirus subgroups, the lowest incidence of COVID-19 was found in the OC43-infected subgroup with 5.2%, which was lower than that of Cohort 2 (Table 2).

The day 30 mortality rate of Cohort 1 and Cohort 2 due to COVID-19 did not differ significantly (3.7% [n = 1/27] in Cohort 1 vs. 4.2% [n = 1/24] in Cohort 2 [p = 0.932]), one patient died on day 1 of the COVID-19 diagnosis and the other on day 6 of COVID-19 diagnosis (Table 1).

When we examined the possible effect of the recent HCoV infection on COVID-19, we found that the incidence was 11% (12/109) and not lower in the subgroup of HCoV cases from the period of March 2019 to March 2020. Furthermore, patients infected by HCoV during the most recent 3-month and 6-month period before March 2020 were also not low i.e. 11.4% (4/35) in the last 3-month and 13.4% (9/67) in the last 6-month subgroup (Table 3). We did not find any significant difference (p > 0.05) in the rates of COVID-19 contraction between individuals who had a recent HCoV infection and their matched control cases (Table 3). Finally, since the lowest COVID-19 incidence was in the OC43 HCoV-infected subgroup, we analyzed the effect of recent infection on COVID-19. COVID-19 incidence was 0/4, 0/9, and 1/12 in the subgroup infected with OC43 during the previous 3-, 6-, and 12-month periods (comparisons with the incidences of the subgroup infected with other HCoV did not reveal significant differences [p = 0.475, p = 0.241, p = 0.776]).

## 4. Discussion

In this study, we analyzed whether HCoV, which acts through the same receptors and defense mechanisms and has a certain level of genetic or antigenic similarity [6–8], affects the prevention of COVID-19. However, we determined that previous infection with HCoV was not a protective factor against COVID-19 in our cohort.

Anderson et al. [9] conducted a study to evaluate the relationship between seasonal HCoV antibodies and COVID-19 contraction. HCoV antibodies were detected in most of the 431 samples taken in the prepandemic period, and approximately 20% of these individuals possessed nonneutralizing antibodies that cross-reacted with SARS-CoV-2 spike and nucleocapsid proteins. They reported that samples with prepandemic SARS-CoV-2-reactive antibodies had elevated levels of antibodies against previously circulating betacoronaviruses (especially OC43). However, these antibodies were not associated with protection against SARS-CoV-2 infections or hospitalizations but boosted upon SARS-CoV-2 infection [9].

Similarly, Sagar et al. [10] evaluated the clinical relevance of COVID-19 infection and HCoV infection in 875 previously confirmed HCoV-infected and 15,053 PCR-negative controls. SARS-CoV-2 PCR test was performed in 11.4% (n = 1812) of a total of 15,928 patients, and 25.9% (n = 470) of the tested patients were PCR-positive. Of the SARS-CoV-2 infected patients, 53.6% (n = 252) were hospitalized, and there was no significant difference in the frequency of hospitalization between the HCoV (+) and HCoV (−) groups. When hospitalized HCoV (+) and HCoV (−) patients were evaluated, it was observed that the HCoV (+) group required less intensive care unit stays (OR: 0.1; 95% CI: 0.0–0.7) and a lower need for mechanical ventilators (OR: 0.0; 95% CI: 0.0–1.0). The rates of patients who were hospitalized and died during follow-up were 17.7% in the HCoV (−) group, whereas it was lower with 4.8% in the HCoV (+) group. With these results, unlike our study, they determined that the previously positive group for HCoV was associated with less severe disease and lower mortality rates compared to the HCoV-negative group [10]. However, in our study, we compared the all-cause mortality rates rather than disease severity. We found that there was no significant difference in terms of mortality between Cohort 1 and Cohort 2. Nevertheless, lack of difference may be related to the relatively low numbers in both Cohorts 1 and 2.

Unlike the results of our study, Otlu et al. [11] found a lower incidence of COVID-19 in 64 patients with preexisting HCoV infection compared to the current province (Malatya) incidence during their study period. They used the National COVID-19 surveillance data as we did and showed that four (6.2%) of 64 patients were infected with COVID-19 by the end of 2020, while, simultaneously, the COVID-19 incidence in the province of Malatya ranged from 7.8% (polymerase chain reaction-based diagnosis) to 9.2% (total diagnosis). The differences were reported to be statistically significant (6.2% vs. 7.8%, p < 0.01; 6.2% vs. 9.2%, p < 0.001). In our study, only the OC43 subgroup in Group 1, among all HCoV subgroups, had a COVID-19 incidence of <7.8% (as reported in the Malatya study) [11].

Our study is subject to several limitations. We tried to include all HCoV-positive cases in the study centers after we started using multiplex PCR because the sample size could not be calculated. Since there were no similar studies asking the same research question at the time we planned and collected the study data, we could not calculate the sample size, but a retrospective power analysis revealed the power of the study as 5%, which may be considered low. Thus, our relatively low study sample might have hindered us from demonstrating a potential preventive effect of previous HCoV infection on COVID-19. Nonetheless, given that our data showed a higher incidence of COVID-19 in Cohort 1, the likelihood of such an effect appears to be lower. Unlike COVID-19 tests, the frequency of testing for seasonal coronaviruses, both pre-COVID and during the pandemic, was typically limited in clinical practice due to the unavailability of related kits (mostly because of reimbursement issues) in hospitals. As a result, this might have significantly impacted the homogeneity of patient populations in the HCoV group. Additionally, we were unable to compare our cohorts to the general population as we did not have the exact COVID-19 incidence data for Ankara, İzmir, or Türkiye as a whole as of December 10, 2020. We evaluated the patients in Cohort 1 based on their PCR results related to prepandemic period. It is possible that some patients in Cohorts 1 or 2 were infected with common HCoV before or after their HCoV PCR test results in our study. Furthermore, we could not analyze the presence of serological and cellular immunity related to HCoV or relationship of this immunity with the underlying diseases in Cohorts 1 and 2. This means that patients in Cohort 2, who might have been infected with HCoV, could not be identified. SARS-CoV and MERS-CoV cases, which are among the betacoronaviruses, are very rare in Türkiye [2]. Therefore, the results of these factors are not included among the HCoV positive cases^6^. Another limitation to note is that PCR-negative COVID-19 patients [5] were not included in the study. However, we can confirm that Cohort 1 was infected with a human coronavirus other than SARS-CoV-2, and their COVID-19 infection status was analyzed using the most commonly used method worldwide, which is PCR. Despite these limitations, this study is one of the few that investigates whether a prior HCoV infection reduces the risk of contracting COVID-19 [12]. Data were collected from three major university hospitals in two major Turkish provinces. To our knowledge, this is the most comprehensive study on this topic in Türkiye, a country that had one of the highest numbers of COVID-19 cases globally during the study period. The study also examined the relationship between HCoV and COVID-19, including its subgroups, and the impact of the near-term immunological response.

In conclusion our data suggest that being previously infected with HCoV did not provide protection against COVID-19 in our study group. We suggest evaluating the possible effect of previous OC43 infection on COVID-19 contraction in larger cohorts.

## Figures and Tables

**Figure:**
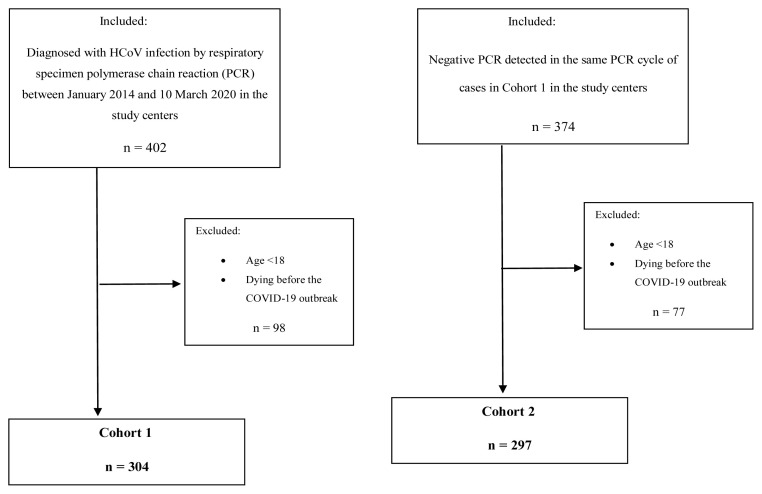
Study inclusion and exclusion criteria and study population.

**Table 1:** Comparison of COVID-19 incidence in the study cohorts (as of December 10, 2020).

	Cohort 1 (n = 304)	Cohort 2 (n = 297)	p
Female	159 (52.3%)	145 (48.8%)	0.393
Male	145 (47.7%)	152 (51.2%)	
Age	47.97 ± 18.13	49.96 ± 19.03	0.189
COVID-19 positivity (as of December 10, 2020)	27 (8.9%)	24 (8.1%)	0.724
The day 30 mortality rate due to COVID-19	1/27 (3.7%)	1/24 (4.2%)	0.932

**Table 2:** Comparison of COVID-19 incidence in the Cohort 1 subgroups (as of December 10, 2020).

		COVID-19-negative	COVID-19-positive	p
Alfacoronavirus (61.8%)		171/188 (90.9%)	17/188 (9.1%)	0.822
Betacoronavirus (31.9%)		89/97 (91.8%)	8/97 (8.2%)	
Nonsubgroup (6.3%)		17/19 (89.5%)	2/19 (10.5%)	
Alfacoronavirus (n = 188)	229E/NL63	60/63 (95.2%)	3/63 (4.8%)	
	229E	84/92 (91.3%)	8/92 (8.7%)	0.211
	NL63	27/33 (81.8%)	6/33 (18.2%)	
Betacoronavirus (n = 97)	OC43	55/58 (94.8%)	3/58 (5.2%)	
	HKU-1	34/39 (84.6%)	5/39 (15.4%)	

**Table 3:** Comparison of the COVID-19 incidence in the group that contracted HCOV during the period from March 2019 to March 2020 (as of December 10, 2020).

	HCoV-positive	Related controls	p
3-month (December 10, 2021 to March 10, 2022)	4/35 (11.4%)	3/35 (8.6%)	0.690
6-month (September 10, 2019 to March 10, 2022)	9/67 (13.4%)	3/58 (5.2%)	0.117
12-month (March 10, 2019 to March 10, 2020)	12/109 (11%)	6/97 (6.2%)	0.221

## Footnotes

1 STROBE (2023). Checklist [online] Website https://www.strobe-statement.org/checklists/ [accessed 20 Aug 2023].

2 Turkish Statistical Institute (TSI) (2021). İstatistiklerle İzmir [online]. Website http://www.izmir.gov.tr/istatistiklerle-izmir [accessed 13 Aug 2021]. (in Turkish)

3 The Governorship of Ankara (2021). İlçelerimiz. [online]. Website http://www.ankara.gov.tr/ilcelerimiz [accessed 13 Aug 2021]. (in Turkish)

4
www.hsys.saglik.gov.tr

5
www.OpenEpi.com

6 European Centre for Disease Prevention and Control (2023) Geographical distribution of confirmed cases of MERS-CoV, by reporting country, April 2012 – June 2023 [online]. Website https://www.ecdc.europa.eu/en/publications-data/geographical-distribution-confirmed-cases-mers-cov-reporting-country-april-2012-7 [accessed 25 Jul 2023]
